# Comparison of the efficacy and safety of denosumab versus bisphosphonates in breast cancer and bone metastases treatment: A meta-analysis of randomized controlled trials

**DOI:** 10.3892/ol.2014.1982

**Published:** 2014-03-20

**Authors:** XIN WANG, KE HU YANG, PINGPING WANYAN, JIN HUI TIAN

**Affiliations:** 1 First Clinical Medical College of Lanzhou University, The First Hospital of Lanzhou University, Lanzhou, Gansu 730000, P.R. China; 2 Evidence-Based Medicine Center, School of Basic Medical Sciences, Lanzhou University, Lanzhou, Gansu 730000, P.R. China; 3 The Second Hospital of Lanzhou University, Lanzhou, Gansu 730000, P.R. China

**Keywords:** denosumab, bisphosphonates, meta-analysis, breast cancer, bone metastases

## Abstract

Breast cancer is the most common type of cancer in females worldwide. Patients with breast cancer and bone metastases may experience increased osteoclast activity, resulting in local bone destruction and skeletal complications, including pain, hypercalcemia and skeletal-related events. Intravenous bisphosphonates (BPs) are the standard treatment administered to patients with breast cancer and bone metastases to prevent skeletal-related events. However, in certain patients, BPs may cause renal toxicity, acute-phase reactions and osteonecrosis of the jaw. More effective, safer and more tolerable therapies, which prevent bone destruction and skeletal complications, are required in order to improve patient quality of life. Denosumab is a fully human monoclonal antibody that binds to and neutralizes receptor activator of nuclear factor-κB ligand, which is a key mediator in the pathogenesis of a broad range of skeletal diseases, thereby inhibiting osteoclast function and bone resorption. Therefore, we conducted a meta-analysis to compare both the safety and efficacy of denosumab and BPs in the treatment of breast cancer and bone metastases. Five databases, two clinical trial registry platforms and reference lists of relevant papers were analyzed. The meta-analysis concluded that denosumab was more effective at preventing pain and skeletal-related events than BPs, in patients with breast cancer and bone metastases. Patients receiving denosumab demonstrated a higher level of clinical improvement in terms of health-related quality of life than patients receiving BPs. Compared with BPs, denosumab reduced the incidence of certain indicators of adverse events, including pyrexia, bone pain, edema and renal failure.

## Introduction

Breast cancer is the most common type of cancer in females worldwide (135 countries), both in developing and developed countries. There are ~1.38 million new cases and 458,000 mortalities caused by breast cancer each year (1). More than 209,000 new cases of breast cancer are expected annually in the United States (2). Bone metastases arises in 70–80% of patients with advanced breast cancer (3). In America, approximately 69–73% of patients with breast cancer, when examined postmortem, exhibit evidence of bone metastases (4).

Patients with breast cancer and bone metastases may experience increased osteoclast activity, resulting in local bone destruction and skeletal complications, which include pain, hypercalcemia and skeletal-related events (SREs) (5–7). Elevated levels of bone turnover markers, such as urine N-telopeptide, represent excessive levels of bone resorption and predict an increased risk of skeletal complications, which may lead to disease progression and mortality (8–10). Pain resulting from bone metastases, in patients with breast cancer, may cause an additional emotional and physical burden for those patients (11,12). Hypercalcemia in malignancy is a reversible but potentially life-threatening consequence of advanced disease (2). SREs, such as fracture and spinal cord compression, are associated with poorer physical, functional and emotional status, lower overall quality of life (13,15) and reduced survival in cancer patients (16,17).

Alleviation of pain and the prevention of bone destruction and SREs can improve the physical, emotional, functional and social aspects of life (18–20), improving overall quality.

Intravenous (IV) bisphosphonates (BPs), such as pamidronate (21) and zoledronic acid (ZA) (22), which are a standard treatment for patients with breast cancer and bone metastases, are effective at preventing the occurrence of SREs (23–26). Although IV BPs, such as ZA, pamidronate, and ibandronate can be effective in the treatment of complications caused by bone metastases, bone destruction and skeletal complications still occur in a large proportion of patients. BPs may cause renal toxicity (27,28), acute-phase reactions (29), osteonecrosis of the jaw (ONJ) and IV administration (30). More effective, safer, more tolerable therapies are required to prevent bone destruction and skeletal complications to improve patient quality of life.

Receptor activator of nuclear factor-κB ligand (RANKL) is a key mediator in the pathogenesis of a broad range of skeletal diseases. In particular, elevated RANKL expression is exhibited in patients with breast cancer (31). Denosumab, approved by the FDA (32), is a fully human monoclonal antibody that binds to and neutralizes RANKL, thereby inhibiting osteoclast function and bone resorption. It is administered as a subcutaneous injection and is not excreted through the kidney; a potential advantage when compared with BPs, for patients with chronic kidney disease.

The present meta-analysis was conducted in order to investigate the efficiency and safety of both denosumab and BPs in patients with bone metastases as a result of breast cancer.

## Materials and methods

### Search strategy

All relevant published randomized controlled trials (RCTs) up to 1 June 2013, were identified. The selected RCTs compared Denosumab to any intervention for breast cancer and bone metastases. PubMed (1966–2013.06), the Cochrane Library (issue 3, 2012), Embase (1974–2013.06), Science Citation Index (1970–2013.06), the Chinese Biomedical Literature Database (1978–2013.06), International Clinical Trials Registry Platform and the Chinese Clinical Trial Register were searched using the following Medical Subject Headings or phrases: Breast neoplasms, breast cancer, neoplasm metastasis, bone metastasis, denosumab, Xgeva, PROLIA, randomized controlled trial and clinical trial.

### Inclusion criteria

RCTs that compared denosumab to any intervention for breast cancer and bone metastases were considered eligible. The selected RCTs met the following criteria: i) participants ≥18 years old; ii) patients had not previously received IV BPs; iii) patients with histologically or cytologically confirmed breast adenocarcinoma with at least one bone metastasis; and iv) patients reported at least one of the following results: SRE, overall survival, percentage reduction in bone turnover markers or adverse events (AEs).

### Exclusion criteria

Trials whereby patients had experienced prior treatment with IV BPs were excluded.

### Outcome measure

SREs and overall survival time were considered as the primary outcome. SREs included fracture, spinal cord compression, hypocalcaemia, radiation to the bone, bone surgery and hypercalcemia in malignancy. Overall survival time was measured as the time period between the point of entering into RCT and mortality. The secondary outcomes studied were pain and AEs. Pain outcomes were assessed by time-to-event and responder analyses. The brief pain inventory-short form (BPI-SF) scores pain severity on a scale from 0 to 10, where 0 represents ‘no pain’ and 10 represents ‘as severe a pain as the patient can imagine’ (33). Pain endpoints included: i) worsening or improvement in pain severity, as measured by the time to an increase or a decrease of 2 points in the pain severity score from baseline and the proportion of patients experiencing an increase or a decrease of 2 points in pain severity; ii) a delay in pain progression, as measured by the time to moderate or severe pain (score, >4 points) among patients who had no or mild pain (score, 0–4 points) at baseline and the proportion of patients experiencing moderate or severe pain among patients who had no or mild pain at baseline; iii) an increase or a decrease in pain interference, as measured by the time to an increase or a decrease of 2 points in the pain interference score from baseline; iv) the time to an increase of 2 points in pain interference among patients who had no or mild pain at baseline; and v) increased analgesic use, as measured by the time to use of strong opioid analgesics and the proportion of patients requiring strong opioid. AEs refer to symptoms or disease caused by therapy. Any outcomes were considered when the information was available.

### Data collection and analysis

Two reviewers screened all titles, abstracts and full text independently to identify citations which matched the selection criteria. Disagreements were resolved by discussion. The following raw data were extracted: Number of patients, age, follow-up year, primary patient diagnosis, number of SREs, overall survival time, number of complications and type of medicine.

### Assessment of methodological quality

The quality of the included RCTs was assessed using the Cochrane Handbook for Systematic Reviews of Interventions, Version 5.1.0 (34). The following factors were assessed for risk of bias in each study: Generation of the randomization sequence, allocation concealment, blinding method, incomplete outcome data, selective outcome reporting and other sources of bias. All items were rated as either at low, unclear or high risk of bias.

### Statistical analysis

For dichotomous outcome results, relative odds ratio (OR) and 95% confidence intervals (CIs) were calculated. For quantitative outcome data, mean differences and 95% CIs were calculated.

Data were analyzed using Review Manager (version 5.1; http://tech.cochrane.org/revman). A P-value of <0.10 was considered to indicate a statistically significant difference and the I^2^ statistic was measured to evaluate statistical heterogeneity among studies. When the P-value was <0.10 and the I^2^ value was >50%, showing heterogeneity, a fixed-effect model was not suitable and a random-effects model was applied. Sensitive analysis was applied to studies that may have affected the outcomes of the meta-analysis. In this article, sensitive analysis means by excluding studies to observe whether there are changes in the statistical results. The sensitive analyses that are done do not materially change the results and it strengthens the confidence that can be placed in these results.

## Results

### Screening outcome

**Figure 1:**
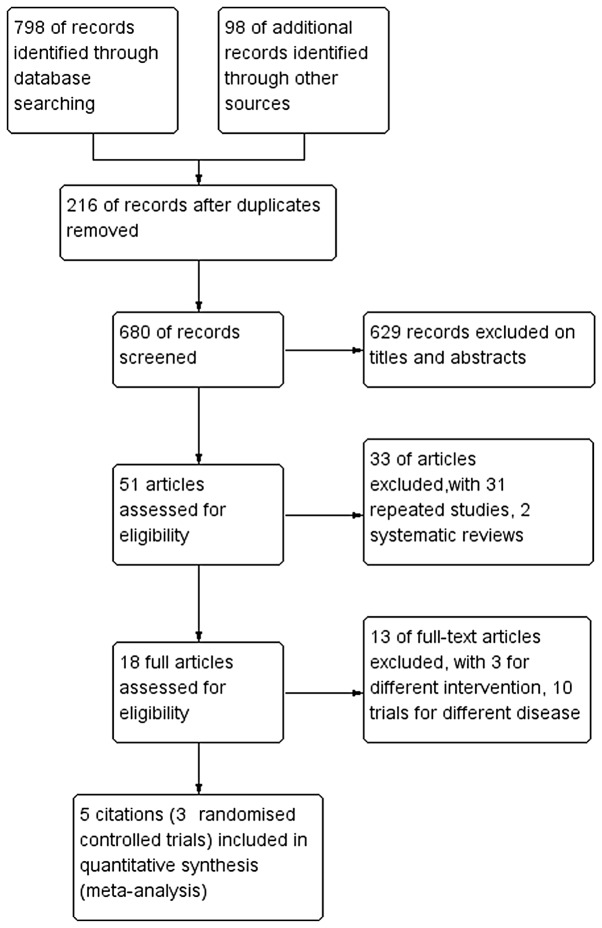
Flow diagram used for the selection process.

Fig. 1 shows the flow chart used for the selection process. After each publication was reviewed, three trials’, five RCTs’ (34–38), included RCT in our study met the inclusion criteria, including a total of 2,330 patients. All included studies were published in English between 2008 and 2013.

### Characteristics

Table I contains basic information obtained from the included studies, such as mean age, number of patients, interventions, outcome and study duration. Three trials (36–38) were from the same study.

### Quality assessment outcome

**Figure 2:**
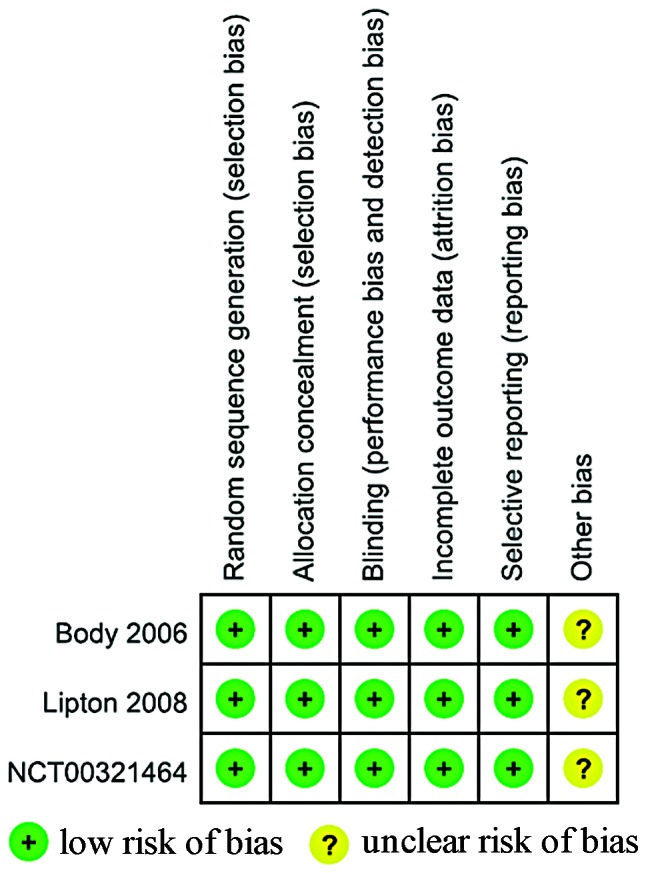
Risk of bias summary.

Fig. 2 demonstrates the process used to assess methodological quality, as used in the Cochrane Handbook, Version 5.1.0. All trials were described as randomized, double-blind, double-dummy, active-controlled, multicenter studies. Patients, investigators and staff were blinded to treatment assignments. The incomplete outcome data (likely to be related to true outcome, with either imbalance in numbers or reasons for missing data across intervention groups) and selective outcome reporting were rated as ‘Yes’. All other sources of bias were rated as ‘unclear’, as there was insufficient information available for a suitable judgment to be made.

### Incidence of SREs

There was no heterogeneity between the studies (P=0.79; I^2^=0%). Comparison of denosumab and BPs (including ZA, pamidronate or ibandronate) for treating breast cancer and bone metastases demonstrated that there was a statistically significant difference in the incidence of SREs (OR 0.61; 95% CI, 0.51–0.72) with the fixed-effect model (Table II).

### Overall survival time

One trial (36) reported overall survival times. There was no significant difference identified between denosumab- and ZA-treated groups (HR, 0.95; 95% CI, 0.81–1.11; P=0.49) and disease progression was similar between the two groups (HR, 1.00; 95% CI, 0.89–1.11; P=0.93)

### AEs

Table II shows the AEs. Three trials (35–37) reporting AEs were identified in the two groups. Excluding arthralgia, anemia and dyspnea, AEs showed homogeneity (I_2_<50%). Incidence of pyrexia, bone pain and edema in all AEs was identified to be significantly different between the denosumab- and BP-treated groups. (P<0.05). A statistically significant difference in the incidence of ONJ was not observed between the denosumab-treated (20/1020) and ZA-treated (14/1013) groups (P=0.39) (36). ONJ was not identified in Denosumab or BP groups in another trial (35).

### Pain

One trial (39) assessed pain outcomes, including variation in pain severity, delay in pain progression, an increase or decrease in pain interference and the time taken for an increase of ≥2 points to be observed in pain interference scores among patients who had no or mild pain at baseline. Results revealed that denosumab-treated patients exhibited a lower incidence of worsening pain severity (2-point increase from baseline) than ZA-treated patients. When analyzing the median time elapsed prior to pain worsening, an increase was observed with denosumab (8.5 months) when compared with ZA (7.4 months) (HR, 0.90; 95% CI, 0.80–1.01; P=0.08). Patients treated with denosumab, who had no or mild pain at baseline, experienced a 4-month delay in median time taken for pain to worsen to moderate or severe score, when compared with ZA-treated patients (denosumab, 9.7 months; ZA, 5.8 months; P=0.002). There was no significant difference in the median time elapsed prior to meaningful pain improvement (defined as a change of 2 points) between the groups (denosumab, 2.7 months; ZA, 2.8 months; HR, 1.02; 95% CI, 0.91–1.15; P=0.72). There was also no statistically significant difference in the time taken for an increase in aggregate pain interference to be observed (≥2 points from baseline; denosumab, 16.0 months; ZA, 14.9 months; HR, 0.89; 95% CI, 0.78–1.02; P=0.90) or in the time taken for a meaningful decrease in aggregate pain interference to be observed (≥2 points from baseline) (denosumab, 2.9 months; ZA, 3.2 months; HR, 0.99; 95% CI, 0.86–1.15; P=0.92).

### Health-related quality of life (HRQL)

During 18 months, a clinically meaningful improvement in HRQL, defined as a ≥5-point change from baseline, on assessment of a general cancer therapy questionnaire (40), was reported in one trial (38). In the present study, an average of 10% more patients treated with denosumab experienced a clinical improvement in HRQL compared with those treated with ZA.

## Discussion

Breast cancer is one of the most common causes of cancer-related mortality worldwide. It commonly affects females aged between 45 and 55 years old. Bone is the most prevalent site for distant spread of breast cancer, with more than half of females with metastatic breast cancer experiencing bone metastases (4). Approximately two-thirds of patients with breast cancer and bone metastasis experience SREs (41), which cause bone pain. SREs and pain can severely affect quality of life and survival of cancer patients.

The purpose of treatment is to delay the progression of bone metastases and enhance the patient’s quality of life and survival. Several placebo-controlled trials have demonstrated that BP therapy with zoledronic acid, pamidronate, clodronate and ibandronate can block the progression of tumor cells in the bone, leading to markedly fewer bone lesions and bone fractures in patients (42–44). However BP use has limitations: i) IV administration is required and ii) BPs may potentially cause serious adverse effects, such as renal toxicity and ONJ (45).

Therefore, more effective, safer treatments are required. Denosumab is a fully human monoclonal antibody against the receptor activator of nuclear factor-κB ligand (RANKL), a cytokine that is essential for the formation, function, and survival of osteoclasts. By binding RANKL, denosumab prevents osteoclast-mediated bone destruction (31,32).

The present meta-analysis was conducted with the aim of investigating the efficacy and safety of denosumab among patients with breast cancer and bone metastases. SREs were the major focus of this meta-analysis in which the following indicators were combined as SREs: Any pathological fracture, a requirement for surgical intervention and palliative radiotherapy to bone lesions, hypercalcemia in malignancy and spinal cord compression. The current study demonstrated that denosumab was more effective than BP therapy at preventing SREs in patients with breast cancer and bone metastases. It was also revealed that overall survival in the BP group was similar to that in the denosumab group. No significant difference was identified between the two groups for certain AEs, which included the following indicators: Nausea, fatigue, arthralgia, back pain, vomiting, anemia, diarrhea, dyspnea, pain in extremity, headache, constipation, asthenia, cough and renal failure. However, patients with breast cancer that had metastasized to the bone, who were receiving denosumab, had a significantly lower incidence of pyrexia, bone pain and edema than those treated with BPs. Compared with BP treatment, more patients in the denosumab group compared with the BP group had a pain prevention and comparable pain palliation and clinically meaningful improvement in HRQoL.

This meta-analysis included five studies, which were all RCTs, three of which reported different indicators from one trial. All the studies evaluated were multicenter, randomized, double-blind (patients and evaluators), double-dummy trials with clear inclusion/exclusion criteria. All RCTs had comprehensive description of baseline (age, gender and average duration) and described loss to exit the situation. As a result of limited communication channels, a small amount of non-English literature may have been missed. The aforementioned factors may affect the evaluation findings and clinical applicability to a certain extent.

This study contains several other potential limitations: i) although a detailed search strategy was developed, there may still be undetected research; ii) the number of included studies is relatively small; iii) in addition to the use of uniform indicators of SREs and AEs, other observed indicators were less consistent in description and a number of indicators, such as overall survival and pain prevention, were not reported in all trials. All of these factors may affect the strength of the conclusions that have been extrapolated from the meta-analysis.

However, the large sample size (>2,000) and homogeneity of included studies still allowed a conclusion to be made. The findings of this study contribute to growing evidence which suggests that denosumab is more effective than BPs at preventing SREs and pain in patients with breast cancer and bone metastases. Patients receiving denosumab exhibited a higher clinical improvement in HRQL scores and, when compared with BPs, denosumab is considered to be more effective at reducing the incidence of certain indicators of AEs, such as pyrexia, bone pain, edema and renal failure.

## Figures and Tables

**Table I:** Basic information from the studies.

First author, year (ref.)	No. of patients		Mean age (years)		Intervention		Follow-up (months)	Outcomes
		
	Denosumab	BP	Denosumab	BP	Denosumab	BP		
Lipton, 2008 (35)	212	43	58	52	Denosumab	Zoledronic acid, pamidronate, ibandronate	57	SREs, NTx, AEs
Body, 2006 (36)	24	5	56	59	Denosumab	Pamidronate	12	SREs, NTx, AEs
Stopeck, 2010 (37)	1026	1020	57	56	Denosumab	Zoledronic acid	136	SREs, AEs, OS
Martin, 2012 (38)	1026	1020	57	56	Denosumab	Zoledronic acid	136	HRQL
Cleeland, 2013 (39)	1026	1020	57	56	Denosumab	Zoledronic acid	136	Pain outcomes

BP, bisphosphonate; SREs, skeletal-related events; NTx, the percentage change from baseline in urine N-telopeptide; AEs, adverse events; OS, overall survival; HRQL, health-related quality of life.

**Table II:** Main outcomes.

		n (events/total events)		Heterogeneity		Effect estimate		
					
Outcomes	Study	Denosumab	BP	I^2^ (%)	P-value	OR	95% CI	P-value
SRE	(35, 37)	496/1238	602/1063	0	0.79	0.61	0.51–0.72	0.00
Adverse events								
Nausea	(35, 37)	403/1231	394/1056	0	0.86	0.88	0.74–1.05	0.16
Fatigue	(35, 37)	335/1255	331/1061	0	0.75	0.89	0.74–1.08	0.24
Arthralgia	(35, 37)	274/1231	304/1056	83	0.01	0.53	0.20–1.39	0.19
Back pain	(35, 37)	271/1231	268/1056	13	0.28	0.90	0.74–1.09	0.29
Pyrexia	(35, 37)	188/1231	256/1056	33	0.22	0.60	0.49–0.75	0.00
Bone pain	(35, 37)	212/1231	246/1013	0	0.72	0.72	0.58–0.89	0.00
Vomiting	(35, 37)	248/1231	246/1056	0	0.91	0.86	0.70–1.05	0.14
Anemia	(35, 37)	215/1231	234/1056	57	0.13	1.10	0.39–3.13	0.86
Diarrhea	(35, 37)	266/1231	214/1056	0	0.82	1.13	0.92–1.39	0.23
Dyspnea	(35, 37)	234/1231	195/1056	65	0.09	0.87	0.35–2.13	0.76
Pain in extremity	(35, 37)	225/1231	230/1056	42	0.19	0.87	0.70–1.07	0.17
Headache	(35, 37)	225/1231	222/1056	0	0.53	0.88	0.71–1.09	0.23
Constipation	(35, 37)	202/1231	212/1056	0	0.79	0.82	0.66–1.01	0.07
Edema	(35, 37)	36/1231	46/1056	0	0.73	0.52	0.32–0.83	0.00
Asthenia	(37)	34/211	12/43	-	-	0.50	0.23–1.06	0.07
Cough	(37)	18/211	7/43	-	-	0.48	0.19–1.23	0.13
Renal failure	(37)	2/1020	25/1013	-	-	0.08	0.02–0.33	0.00
ONJ	(37)	20/1020	14/1013	-	-	1.43	0.72, 2.84	0.31

BP, bisphosphonate; SREs, skeletal-related events; OR, odds ratio; CI, confidence interval; ONJ, osteonecrosis of the jaw.

## References

[1] Bray F, Jemal A, Grey N, Ferlay J, Forman D (2012). Global cancer transitions according to the Human Development Index (2008–2030): a population-based study. Lancet Oncol.

[2] Coleman RE (2006). Clinical features of metastatic bone disease and risk of skeletal morbidity. Clin Cancer Res.

[3] Coleman RE, Lipton A, Roodman GD, Guise TA, Boyce BF, Brufsky AM, Clézardin P, Croucher PI, Gralow JR, Hadji P (2010). Metastasis and bone loss: advancing treatment and prevention. Cancer Treat Rev.

[4] Coleman RE, Rubens RD (1987). The clinical course of bone metastases from breast cancer. Br J Cancer.

[5] Coleman R (2007). Potential use of bisphosphonates in the prevention of metastases in early-stage breast cancer. Clin Breast Cancer.

[6] Body JJ (2003). Effectiveness and cost of bisphosphonate therapy in tumor bone disease. Cancer.

[7] Coleman RE (2001). Metastatic bone disease: clinical features, pathophysiology and treatment strategies. Cancer Treat Rev.

[8] Brown JE, Cook RJ, Major P, Lipton A, Saad F, Smith M, Lee KA, Zheng M, Hei YJ, Coleman RE (2005). Bone turnover markers as predictors of skeletal complications in prostate cancer, lung cancer, and other solid tumors. J Natl Cancer Inst.

[9] Costa L, Demers LM, Gouveia-Oliveira A, Schaller J, Costa EB, de Moura MC, Lipton A (2002). Prospective evaluation of the peptide-bound collagen type I cross-links N-telopeptide and C-telopeptide in predicting bone metastases status. J Clin Oncol.

[10] Coleman RE, Major P, Lipton A, Brown JE, Lee KA, Smith M, Saad F, Zheng M, Hei YJ, Seaman J, Cook R (2005). Predictive value of bone resorption and formation markers in cancer patients with bone metastases receiving the bisphosphonate zoledronic acid. J Clin Oncol.

[11] Nørgaard M, Jensen AØ, Jacobsen JB, Cetin K, Fryzek JP, Sørensen HT (2010). Skeletal related events, bone metastasis and survival of prostate cancer: a population based cohort study in Denmark (1999 to 2007). J Urol.

[12] Sathiakumar N, Delzell E, Morrisey MA, Falkson C, Yong M, Chia V, Blackburn J, Arora T, Kilgore ML (2011). Mortality following bone metastasis and skeletal-related events among men with prostate cancer: a population-based analysis of U.S. Medicare beneficiaries, 1999–2006. Prostate Cancer Prostatic Dis.

[13] Costa L, Badia X, Chow E, Lipton A, Wardley A (2008). Impact of skeletal complications on patients’ quality of life, mobility, and functional independence. Support Care Cancer.

[14] Costa L, Major PP (2009). Effect of bisphosphonates on pain and quality of life in patients with bone metastases. Nat Clin Pract Oncol.

[15] Pockett RD, Castellano D, McEwan P, Oglesby A, Barber BL, Chung K (2010). The hospital burden of disease associated with bone metastases and skeletal-related events in patients with breast cancer, lung cancer, or prostate cancer in Spain. Eur J Cancer Care (Engl).

[16] Oefelein MG, Ricchiuti V, Conrad W, Resnick MI (2002). Skeletal fractures negatively correlate with overall survival in men with prostate cancer. J Urol.

[17] Saad F, Lipton A, Cook R, Chen YM, Smith M, Coleman R (2007). Pathologic fractures correlate with reduced survival in patients with malignant bone disease. Cancer.

[18] Weinfurt KP, Castel LD, Li Y, Timbie JW, Glendenning GA, Schulman KA (2004). Health-related quality of life among patients with breast cancer receiving zoledronic acid or pamidronate disodium for metastatic bone lesions. Med Care.

[19] Wardley A, Davidson N, Barrett-Lee P, Hong A, Mansi J, Dodwell D, Murphy R, Mason T, Cameron D (2005). Zoledronic acid significantly improves pain scores and quality of life in breast cancer patients with bone metastases: a randomised, crossover study of community vs hospital bisphosphonate administration. Br J Cancer.

[20] Abrahm JL, Banffy MB, Harris MB (2008). Spinal cord compression in patients with advanced metastatic cancer: “all I care about is walking and living my life. JAMA.

[21] Theriault RL, Lipton A, Hortobagyi GN, Leff R, Glück S, Stewart JF, Costello S, Kennedy I, Simeone J, Seaman JJ (1999). Pamidronate reduces skeletal morbidity in women with advanced breast cancer and lytic bone lesions: a randomized, placebo-controlled trial. J Clin Oncol.

[22] Rosen LS, Gordon D, Tchekmedyian S, Yanagihara R, Hirsh V, Krzakowski M, Pawlicki M, de Souza P, Zheng M, Urbanowitz G, Reitsma D, Seaman JJ (2003). Zoledronic acid versus placebo in the treatment of skeletal metastases in patients with lung cancer and other solid tumors: a phase III, double-blind, randomized trial - the Zoledronic Acid Lung Cancer and Other Solid Tumors Study Group. J Clin Oncol.

[23] Aapro M, Abrahamsson PA, Body JJ, Coleman RE, Colomer R, Costa L, Crinò L, Dirix L, Gnant M, Gralow J (2008). Guidance on the use of bisphosphonates in solid tumours: recommendations of an international expert panel. Ann Oncol.

[24] Hillner BE, Ingle JN, Chlebowski RT, Gralow J, Yee GC, Janjan NA, Cauley JA, Blumenstein BA, Albain KS, Lipton A, Brown S, American Society of Clinical Oncology (2003). American Society of Clinical Oncology 2003 update on the role of bisphosphonates and bone health issues in women with breast cancer. J Clin Oncol.

[25] Basch EM, Somerfield MR, Beer TM, Carducci MA, Higano CS, Hussain MH, Scher HI, American Society of Clinical Oncology (2007). American Society of Clinical Oncology endorsement of the Cancer Care Ontario Practice Guideline on nonhormonal therapy for men with metastatic hormone-refractory (castration-resistant) prostate cancer. J Clin Oncol.

[26] Rosen LS, Gordon D, Kaminski M, Howell A, Belch A, Mackey J, Apffelstaedt J, Hussein MA, Coleman RE, Reitsma DJ (2003). Long-term efficacy and safety of zoledronic acid compared with pamidronate disodium in the treatment of skeletal complications in patients with advanced multiple myeloma or breast carcinoma: a randomized, double-blind, multicenter, comparative trial. Cancer.

[27] Markowitz GS, Fine PL, Stack JI, Kunis CL, Radhakrishnan J, Palecki W, Park J, Nasr SH, Hoh S, Siegel DS, D’Agati VD (2003). Toxic acute tubular necrosis following treatment with zoledronate (Zometa). Kidney Int.

[28] Perazella MA, Markowitz GS (2008). Bisphosphonate nephrotoxicity. Kidney Int.

[29] Novartis Pharmaceuticals Corporation (2011). Zometa (zoledronic acid) prescribing information.

[30] Mauri D, Valachis A, Polyzos IP, Polyzos NP, Kamposioras K, Pesce LL (2009). Osteonecrosis of the jaw and use of bisphosphonates in adjuvant breast cancer treatment: a meta-analysis. Breast Cancer Res Treat.

[31] Thomas RJ, Guise TA, Yin JJ, Elliott J, Horwood NJ, Martin TJ, Gillespie MT (1999). Breast cancer cells interact with osteoblasts to support osteoclast formation. Endocrinology.

[32] US Food and Drug Administration (2010). Denosumab (Xgeva, Amgen) approval for the prevention of skeletal-related events in patients with bone metastases from solid tumors.

[33] Serlin RC, Mendoza TR, Nakamura Y, Edwards KR, Cleeland CS (1995). When is cancer pain mild, moderate or severe? Grading pain severity by its interference with function. Pain.

[34] Higgins JPT, Green S (2011). Cochrane Handbook for Systematic Reviews of Interventions Version 5.1.0.

[35] Lipton A, Steger GG, Figueroa J, Alvarado C, Solal-Celigny P, Body JJ, de Boer R, Berardi R, Gascon P, Tonkin KS (2008). Extended efficacy and safety of denosumab in breast cancer patients with bone metastases not receiving prior bisphosphonate therapy. Clin Cancer Res.

[36] Body JJ, Facon T, Coleman RE, Lipton A, Geurs F, Fan M, Holloway D, Peterson MC, Bekker PJ (2006). A study of the biological receptor activator of nuclear factor-kappaB ligand inhibitor, denosumab, in patients with multiple myeloma or bone metastases from breast cancer. Clin Cancer Res.

[37] Stopeck AT, Lipton A, Body JJ, Steger GG, Tonkin K, de Boer RH, Lichinitser M, Fujiwara Y, Yardley DA, Viniegra M (2010). Denosumab compared with zoledronic acid for the treatment of bone metastases in patients with advanced breast cancer: a randomized, double-blind study. J Clin Oncol.

[38] Martin M, Bell R, Bourgeois H, Brufsky A, Diel I, Eniu A, Fallowfield L, Fujiwara Y, Jassem J, Paterson AH (2012). Bone-related complications and quality of life in advanced breast cancer: results from a randomized phase III trial of denosumab versus zoledronic acid. Clin Cancer Res.

[39] Cleeland CS, Body JJ, Stopeck A, von Moos R, Fallowfield L, Mathias SD, Patrick DL, Clemons M, Tonkin K, Masuda N (2013). Pain outcomes in patients with advanced breast cancer and bone metastases: results from a randomized, double-blind study of denosumab and zoledronic acid. Cancer.

[40] Webster K, Cella D, Yost K (2003). The functional assessment of chronic illness therapy (FACIT) measurement system: properties, applications, and interpretation. Health Qual Life Outcomes.

[41] Coleman RE (1997). Skeletal complications of malignancy. Cancer.

[42] Kristensen B, Ejlertsen B, Groenvold M, Hein S, Loft H, Mouridsen HT (1999). Oral clodronate in breast cancer patients with bone metastases: a randomized study. J Intern Med.

[43] Body JJ, Lichinitser M, Tjulandin S, Garnero P, Bergström B (2007). Oral ibandronate is as active as intravenous zoledronic acid for reducing bone turnover markers in women with breast cancer and bone metastases. Ann Oncol.

[44] Kohno N, Aogi K, Minami H, Nakamura S, Asaga T, Iino Y, Watanabe T, Goessl C, Ohashi Y, Takashima S (2005). Zoledronic acid significantly reduces skeletal complications compared with placebo in Japanese women with bone metastases from breast cancer: a randomized, placebo-controlled trial. J Clin Oncol.

[45] Olson K, Van Poznak C (2007). Significance and impact of bisphosphonate-induced acute phase responses. J Oncol Pharm Pract.

